# From Positive Youth Development to Positive Aging: A Case on Prosociality

**DOI:** 10.1093/geroni/igaf122.1878

**Published:** 2025-12-31

**Authors:** Dwight Tse

**Affiliations:** University of Strathclyde, Glasgow, Scotland, United Kingdom

## Abstract

Developmental psychologists often focus research on specific stages across the lifespan, leading to theories that address unique challenges, opportunities, and developmental contexts at each stage. However, this raises the question of whether models designed for one stage can be applied to others. Positive Youth Development (PYD), which emphasizes the potential for growth and positive developmental trajectories in youth, challenges the view of adolescence as a period of “storm and stress.” Similarly, aging has historically been framed as functional decline and frailty, but increasing research highlights the role of prosocial behaviors (e.g., volunteerism) in fostering positive aging outcomes. One prominent PYD theory is Lerner’s 5Cs model (later expanded to 6Cs and 7Cs), identifying caring, confidence, competence, character, and connection as key PYD facilitators. These factors are also linked to prosociality, and growing evidence suggests they play a crucial role in positive aging. In this presentation, I will map how the 5Cs model can be applied in prosocial programs and explore how these same factors are acknowledged in existing aging theories. Using prosociality as an example, I will demonstrate the potential for adopting the 5–7Cs model to the study of positive aging. Finally, I will conclude by discussing the challenges and future directions for studying prosociality in positive aging, within the lifespan perspective, alongside research on positive development at specific life stages.

